# Species Distinction in the Trichophyton rubrum Complex

**DOI:** 10.1128/JCM.00352-19

**Published:** 2019-08-26

**Authors:** Huilin Su, Ann Packeu, Sarah A. Ahmed, Abdullah M. S. Al-Hatmi, Oliver Blechert, Macit İlkit, Ferry Hagen, Yvonne Gräser, Weida Liu, Shuwen Deng, Marijke Hendrickx, Jinhua Xu, Min Zhu, Sybren de Hoog

**Affiliations:** a Department of Dermatology, Huashan Hospital, Fudan University, Shanghai, China; b Center of Expertise in Mycology of Radboud University Medical Center/Canisius Wilhelmina Hospital, Nijmegen, The Netherlands; c Westerdijk Fungal Biodiversity Institute, Utrecht, The Netherlands; d Sciensano, Brussels, Belgium; e Ministry of Health, Directorate General of Health Services, Ibri, Oman; f Department of Mycology, Institute of Dermatology, Chinese Academy of Medical Sciences and Peking Union Medical College, Nanjing, China; g Division of Mycology, Department of Microbiology, Faculty of Medicine, University of Çukurova, Adana, Turkey; h Institut für Mikrobiologie und Hygiene, Charité, Berlin, Germany; i Department of Medical Microbiology, People's Hospital of Suzhou National New & Hi-Tech Industrial Development Zone, Suzhou, China; j Department of Medical Microbiology, University Medical Center Utrecht, Utrecht, The Netherlands; Carter BloodCare & Baylor University Medical Center

**Keywords:** ITS, MALDI-TOF MS, *Trichophyton rubrum* complex, amplified fragment length polymorphisms, dermatophytes, physiology, species distinction

## Abstract

The Trichophyton rubrum species complex comprises commonly encountered dermatophytic fungi with a worldwide distribution. The members of the complex usually have distinct phenotypes in culture and cause different clinical symptoms, despite high genome similarity. In order to better delimit the species within the complex, molecular, phenotypic, and physiological characteristics were combined to reestablish a natural species concept. Three groups, T. rubrum, T. soudanense, and T. violaceum, could be distinguished based on the sequence of the internal transcribed spacer (ITS) ribosomal DNA barcode gene. On average, strains within each group were similar by colony appearance, microscopy, and physiology, but strains between groups showed significant differences. Trichophyton rubrum strains had higher keratinase activity, whereas T. violaceum strains tended to be more lipophilic; however, none of the phenotypic features were diagnostic. The results of matrix-assisted laser desorption ionization–time of flight mass spectrometry (MALDI-TOF MS) and amplified fragment length polymorphism (AFLP) were partially consistent with the ITS data but failed to distinguish the species unambiguously. Despite their close similarity, T. violaceum, T. soudanense, and T. rubrum can be regarded as independent species with distinct geographical distributions and clinical predilections. Trichophyton soudanense is pheno- and genotypically intermediate between T. rubrum and T. violaceum. For routine diagnostics, ITS sequencing is recommended.

## Abstract (precis)

The Trichophyton rubrum species complex comprises commonly encountered dermatophytic fungi with a worldwide distribution. The members of the complex usually have distinct phenotypes in culture and cause different clinical symptoms, despite high genome similarity. In order to better delimit the species within the complex, molecular, phenotypic, and physiological characteristics were combined to reestablish a natural species concept.

## INTRODUCTION

Dermatophytes are keratinophilic fungi that infect mammalian skin, hair, and nails (1). The incidence and prevalence of these superficial infections are extremely high, and it is estimated that over 20 to 25% of the global population is affected (2). The most common dermatophytes belong to the Trichophyton rubrum complex; it comprises phenotypically diverse organisms with distinct clinical, cultural, morphological, and physiological characteristics which were previously known under multiple names, such as T. circonvolutum, T. fischeri, T. fluviomuniense, T. glabrum, T. gourvilii, T. kanei, T. kuryangei, T. megninii, T. pedis, T. raubitschekii, T. rodhainii, T. soudanense, T. violaceum, and T. yaoundei. However, Gräser et al. (3) noticed that the molecular distances between anthropophilic dermatophyte taxa were surprisingly low, and for this reason, most of these names were synonymized with T. rubrum or T. violaceum.

Trichophyton violaceum, the main causative agent of tinea capitis, is a slowly expanding, poorly sporulating organism first described by Sabouraud in 1902. Eight years later, the rapidly growing, abundantly sporulating T. rubrum, responsible for tinea corporis and pedis, was described (4). The majority of species names in the T. rubrum complex were subsequently introduced to describe phenotypic variations. While most of the latter characteristics can now be reevaluated by the use of experimental methodologies, the difference between the preferred infection sites and the concomitant morphological difference between T. rubrum and T. violaceum have remained puzzling. The existence of separate species with different clinical predilections is expected, but an alternative hypothesis is that strains of the single species T. rubrum demonstrate phenotypic differences, i.e., degenerate colony development and increased metabolite production, depending on the infection site.

De Hoog and colleagues have presented an overview of dermatophytes based on the results of multilocus sequencing, including sequencing of the internal transcribed spacer (ITS) ribosomal DNA (rDNA), partial large subunit (LSU), tubulin, and 60S L10 rDNA, which indicated that in molecular phylogeny, T. violaceum is almost indistinguishable from T. rubrum (5, 6). However, that study was focused on defining the main taxonomic trends in dermatophytes and should be supplemented with detailed investigation at the species level. Another study showed that T. rubrum and T. violaceum are in a closely similar ITS1 homology group (7). Gräser et al. (3) evaluated the validity of all taxa with extant type material around T. rubrum by combined morphological, physiological, and molecular methods, including ITS sequencing, PCR fingerprinting, and amplified fragment length polymorphism (AFLP), and confirmed only T. rubrum and T. violaceum as valid species in the complex. Analysis of the T. rubrum diversity with random amplified polymorphic DNA (RAPD) revealed 40 distinct patterns in 55 isolates (8). In PCR fingerprinting, primer (GACA)_4_ was used to amplify sequences of three genera of dermatophytes, producing a species-specific profile for T. rubrum, but the method could not detect differences within the T. rubrum complex (9). T. rubrum was concluded to be clonal after investigation of 96 strains from four continents by PCR fingerprinting, AFLP, and random amplified monomorphic DNA (RAMD) (10), suggesting rapid global spread of the pathogen. A recent study using whole-genome analysis also illustrated the global clonal population structure of T. rubrum (11). Ohst et al. (12) and Gräser et al. (13) detected four polymorphic alleles in T. violaceum and T. rubrum microsatellite marker T1 which were significantly associated with geographical origins. Matrix-assisted laser desorption ionization–time of flight (MALDI-TOF) mass spectrometry (MS) has become popular for routine fungal identification and has an accuracy level of between 13.5 and 100% for dermatophytes (14). Trichophyton violaceum and T. rubrum were also identified with MALDI-TOF MS (15). Packeu et al. (A. Packeu, D. Stubbe, S. Roesems, K. Goens, P. Van Rooij, S. de Hoog, and M. Hendrickx, submitted for publication) included more strains of T. soudanense and distinguished it as a third group according to MALDI-TOF MS and ITS sequencing.

The data presented above demonstrate the apparent molecular variability in the T. rubrum complex, largely corresponding to clinical characteristics and geographical distribution. In an attempt to precisely delimit species within the complex, in the present study we analyzed an expanded data set of strains based on a combination of molecular parameters with ecology and evolution.

## MATERIALS AND METHODS

### Isolates.

Strains were obtained from the reference collections of the Centraalbureau voor Schimmelcultures (CBS; housed at the Westerdijk Fungal Biodiversity Institute, Utrecht, The Netherlands), the Belgian Coordinated Collections of Microorganism-Scientific Institute of Public Health (BCCM/IHEM; Brussels, Belgium), and the Huashan Hospital (Fudan University, Shanghai, China). The isolates were acquired by these collections over several decades. Strain characteristics, including original identification, geographical source, and clinical information, are shown in Table 1.

**TABLE 1:** Strain information, with a summary of ITS, AFLP, and MALDI-TOF MS results and physiological and morphological characteristics^*a*^

ITS	Strain no.	Geography	Source	MALDI-TOF MS result	AFLP pattern	D	Obverse	Reverse	Mi	Ma	Rb	Ur	Ke	Tw
1 H7	CBS 319.31	/	/	T. soudanense	D	23	Cream	Cream	−	−	−	+	−	−
1 H7	CBS 499.48	France	Skin	T. yaoundei	B	15	White	Cream	−	−	−	+	w	−
1 H7	CBS 459.61	Uganda	Scalp	T. violaceum	B	16	Cream	Cream	−	−	−	+	−	−
1 H7	CBS 460.61	Uganda	Scalp	T. yaoundei	C	12	White	Cream	−	−	−	+	−	−
1 H7	CBS 677.82	Morocco	Scalp	T. violaceum	A	12	White	Cream	+	−	−	+	w	35
1 H7	CBS 555.84	Netherlands	Scalp	T. violaceum	A	8	White	Brown	−	−	−	+	w	2
1 H7	CBS 374.92 NT	Netherlands	Skin	T. rubrum	D	10	Cream	Cream	−	−	−	+	w	10
1 H7	CBS 375.92	Netherlands	Skin	*/*	D	9	Cream	Cream-brown	+	−	−	+	+	8
1 H11	CBS 118535	China	Hair	T. soudanense	B	14	White	Cream	−	−	−	+	w	9
1 H7	CBS 120320	Switzerland	Tinea capitis	T. violaceum	D	9	Cream	Brown	−	−	−	+	−	7
1 H7	CBS 141825	China	/	T. soudanense	B	3	Cream	Cream	−	−	−	+	+	7
1 H8	CBS 141826	China	/	T. violaceum	D	7	Cream	Cream	−	−	−	+	w	9
1 H9	CBS 141829	China	/	T. violaceum	C	10	White	Cream	−	−	−	+	w	5
1 H7	IHEM 3663	Belgium	Tinea capitis	T. violaceum	D	15	Cream	Cream	−	−	−	+	−	10
1 H7	IHEM 4887	Belgium	Skin	T. violaceum	B	14	Cream	Cream-brown	−	−	−	+	−	11
1 H7	IHEM 10480	Belgium	Tinea capitis	T. violaceum	D	16	Cream	Cream	−	−	−	+	−	6
1 H7	IHEM 10481	Belgium	Scalp	T. violaceum	D	16	Cream	Cream	−	−	−	+	w	20
1 H10	IHEM 13375	Kenya	Tinea capitis	T. violaceum	A	17	White	White	−	−	−	+	w	8
1 H7	IHEM 13461	Tunesia	Tinea capitis	T. violaceum	D	12	Yellow	Red-brown	−	−	−	+	−	9
1 H7	IHEM 13463	Tunesia	Tinea capitis	T. violaceum	B	8	White	White	−	−	−	w	w	6
1 H7	IHEM 13524	Belgium	Tinea capitis	T. violaceum	D	18	White	White	−	−	−	+	w	8
1 H7	IHEM 13918	Belgium	Human	T. violaceum	D	15	White	White	−	−	−	+	−	5
1 H7	IHEM 15852	Morocco	Tinea capitis	T. violaceum	B	19	Red-brown	Brown	−	−	−	+	−	10
1 H7	IHEM 15898	Congo	E, soil	T. violaceum	D	14	Cream	Cream	−	−	−	+	−	5
1 H7	IHEM 16805	Morocco	Tinea capitis	T. violaceum	D	12	White-brown	White-brown	−	−	−	+	−	12
1 H7	IHEM 17428	Afghanistan	Tinea capitis	T. violaceum	D	13	Cream	Cream	−	−	−	+	−	9
1 H7	IHEM 17829	Belgium	Tinea capitis	T. violaceum	B	12	Cream	Cream-brown	−	−	+	+	+	7
1 H7	IHEM 18680	Belgium	Tinea capitis	T. violaceum	B	12	Yellow	Yellow-brown	−	−	−	w	−	−
1 H7	IHEM 18682	Belgium	Tinea capitis	T. violaceum	D	17	Yellow	Yellow-brown	−	−	−	w	−	7
1 H7	IHEM 18818	Belgium	E, hat	T. violaceum	D	18	Cream	Cream	−	−	+	+	−	5
1 H7	IHEM 19882	Belgium	Tinea capitis	T. violaceum	A	11	Red-brown	Red-brown	−	−	−	w	−	6
1 H7	IHEM 20649	Belgium	Tinea capitis	T. violaceum	A	9	Yellow	Red-brown	+	−	−	+	w	5
1 H7	IHEM 22325	Belgium	Tinea icognito	T. violaceum	B	14	Cream	Cream	−	−	−	+	−	9
1 H7	IHEM 25555	Belgium	Skin	T. violaceum	B	9	White-brown	Yellow-brown	+	−	+	+	−	6
1 H7	IHEM 25578	Iran	Tinea capitis	T. violaceum	B	19	White-brown	Red-brown	−	−	−	+	−	6
1 H7	IHEM 25600	Belgium	Tinea capitis	T. violaceum	B	16	Cream	Cream	−	−	−	+	−	5
1 H7	IHEM 13462	Algeria	Tinea capitis	T. violaceum	D	14	White	Cream	−	−	−	+	−	7
1 H7	IHEM 25601	Netherlands	Tinea capitis	T. violaceum	B	25	White	Cream	+	−	−	+	−	12
1 H7	IHEM 25806	France	/	T. violaceum	D	12	Cream	Cream	−	−	−	+	−	7
1 H7	IHEM 3760	Belgium	Tinea capitis	T. violaceum	D	18	Cream	Cream	+	−	−	+	−	4
1 H7	IHEM 3779	Belgium	Tinea capitis	T. violaceum	B	10	Cream	Cream	−	−	−	+	−	6
1 H7	HS 215-633	China	Hair	T. violaceum	D	7	White	Cream	−	−	−	+	−	8
1 H7	HS 217-1900	China	Hair	T. soudanense	D	10	White	Cream	+	−	−	+	−	3
1 H7	HS 217-1976	China	Hair	T. violaceum	D	14	White-brown	White-brown	−	−	−	+	−	4
1 H7	HS 217-5329	China	Hair	T. violaceum	D	15	White	White-brown	−	−	−	+	+	5
1 H7	CBS 376.92	Netherlands	Skin	T. violaceum	B	3	Brown	Brown	−	−	−	+	w	11
1 H7	CBS 118536	China	/	T. yaoundei	B	15	White	Yellow	−	−	−	+	+	5
1 H7	HS 216-6181	China	Skin	*/*	D	12	White	Cream	−	−	−	+	−	7
1 H12	IHEM 4711	Congo	Tinea capitis	T. yaoundei	D	10	White	White	−	−	−	+	+	2
1 H12	IHEM 13775	Congo	Tinea capitis	T. yaoundei	D	12	White	White	−	−	−	+	w	−
1 H12	IHEM 19041	Belgium	Skin	T. yaoundei	D	18	White	White	−	−	−	+	w	−
1 H12	IHEM 20011	Congo	Tinea capitis	T. yaoundei	B	8	Cream	Cream	−	−	−	+	w	−
1 H12	IHEM 21424	/	/	T. yaoundei	D	12	White	White	−	−	−	+	w	12
1 H12	IHEM 1279	/	/	T. yaoundei	D	17	White	Yellow	+	−	−	+	+	−
1 H12	IHEM 19885	Belgium	Tinea capitis	T. yaoundei	/	10	White	White	−	−	−	+	w	−
1 H12	CBS 305.60^T^	/	/	T. yaoundei	A	16	Cream	Cream	+	−	−	+	+	3
1 H12	CBS 730.88	France	/	T. yaoundei	A	8	White	Yellow	−	−	−	w	−	8
														
2 H2	CBS 286.30	Italy	/	T. soudanense	/	15	White	Yellow	−	−	−	+	w	−
2 H1	CBS 170.65	Nigeria	Skin	T. soudanense	A	20	White	Orange	+	−	+	+	+	−
2 H2	CBS 437.63	Africa	Scalp	T. soudanense	/	16	White	Orange	+	−	+	+	+	−
2 H1	CBS 440.63	Ghana	Scalp	T. soudanense	A	16	White	Yellow-brown	+	−	+	+	w	−
2 H2	CBS 119446	Gabon	Tinea capitis	T. soudanense	A	9	Yellow	Orange	+	−	−	+	+	4
2 H2	CBS 120316	Switzerland	Tinea capitis	T. soudanense	A	10	Yellow	Orange	+	−	+	+	+	−
2 H2	CBS 120317	Switzerland	Tinea capitis	*/*	C	13	Yellow	Orange	−	−	−	+	+	8
2 H2	IHEM 1284	/	/	T. soudanense	A	15	Yellow	Orange	−	−	+	+	w	−
2 H2	IHEM 13459	Somalia	Skin	T. soudanense	A	13	Orange	Orange	+	−	+	+	−	−
2 H2	IHEM 13460	Somalia	Tinea capitis	T. soudanense	C	20	Yellow	Orange	+	−	+	+	−	−
2 H2	IHEM 13521	Somalia	Tinea capitis	T. soudanense	A	16	Yellow	Orange	+	−	+	+	−	−
2 H2	IHEM 13534	Somalia	Tinea capitis	T. soudanense	A	15	White-Yellow	Orange	+	−	+	+	−	−
2 H2	IHEM 16536	Congo	Tinea capitis	T. soudanense	C	12	White	Yellow-Orange	+	−	+	w	+	5
2 H1	IHEM 18812	Belgium	E, cap	T. soudanense	C	18	Yellow	Orange	+	−	+	w	−	−
2 H1	IHEM 18813	Belgium	E, cap	T. soudanense	A	17	Yellow	Orange	+	−	+	+	w	2
2 H2	IHEM 18815	Belgium	Tinea capitis	T. soudanense	C	11	White	Orange	+	−	+	w	+	−
2 H2	IHEM 18919	Belgium	Tinea capitis	T. soudanense	A	11	White	Orange	+	−	+	+	w	2
2 H2	IHEM 19042	Belgium	Tinea capitis	T. soudanense	C	13	White	Orange	+	−	+	+	−	−
2 H2	IHEM 19372	Belgium	Tinea capitis	T. soudanense	A	11	Orange	Orange	+	−	+	w	w	2
2 H2	IHEM 19715	Belgium	Tinea capitis	T. soudanense	A	10	Orange	Orange	+	−	+	+	−	−
2 H2	IHEM 19742	Senegal	Tinea capitis	T. soudanense	A	25	Yellow-orange	Orange-brown	+	−	+	w	−	−
2 H2	IHEM 19743	Senegal	Tinea capitis	T. soudanense	A	14	White	Orange	+	−	+	+	w	7
2 H1	IHEM 19744	Senegal	Tinea capitis	T. soudanense	A	12	White	Yellow-orange	+	−	+	+	−	−
2 H1	IHEM 19746	Ivory Coast	Tinea capitis	T. soudanense	A	14	White	Orange	+	−	+	+	w	−
2 H2	IHEM 19747	Togo	Tinea capitis	T. soudanense	C	14	White	Orange	+	−	+	w	−	−
2 H2	IHEM 19749	USA	/	T. soudanense	C	17	Yellow	Orange	+	−	+	w	−	−
2 H2	IHEM 19750	Ivory Coast	Skin	T. soudanense	C	13	White	Yellow	+	−	+	+	−	−
2 H1	IHEM 19751 NT	Togo	Tinea capitis	T. soudanense	A	17	White	Orange	+	−	+	+	w	−
2 H2	IHEM 19752	Ivory Coast	Tinea capitis	T. soudanense	C	15	White	Orange	+	−	+	+	−	−
2 H1	IHEM 19916	Belgium	E, blanket	T. soudanense	C	10	White	Orange	+	−	+	+	−	−
2 H1	IHEM 19920	Belgium	E, scarf	T. soudanense	C	12	Yellow-orange	Yellow-orange	+	−	+	w	−	−
2 H2	IHEM 19943	Belgium	Tinea capitis	T. soudanense	C	10	Orange	Orange	+	−	+	+	w	−
2 H1	IHEM 20012	Belgium	Tinea capitis	T. soudanense	C	12	White	Orange	+	−	+	+	−	−
2 H2	IHEM 20743	Belgium	E, cushion	T. soudanense	C	13	Yellow	Orange	+	−	+	+	w	−
2 H2	IHEM 20771	Belgium	E, sofa	T. soudanense	C	12	Orange	Orange	+	−	+	+	−	−
2 H2	IHEM 20772	Belgium	Tinea capitis	T. soudanense	C	11	Orange	Orange	+	−	+	w	−	−
2 H2	IHEM 20773	Belgium	Tinea capitis	T. soudanense	C	12	White	Orange	+	−	−	+	−	−
2 H2	IHEM 21666	Belgium	Tinea capitis	T. soudanense	C	18	White	Yellow-orange	+	−	+	+	−	2
2 H2	IHEM 21976	Belgium	Nail	T. soudanense	C	13	Yellow-orange	Orange	+	−	+	w	−	2
2 H2	IHEM 23361	Belgium	Hair	T. soudanense	A	16	White	Orange	+	−	+	+	w	−
2 H1	IHEM 23920	Belgium	Tinea capitis	T. soudanense	A	12	Yellow-orange	Orange	+	−	+	+	−	14
2 H2	IHEM 24425	Belgium	Scalp	T. soudanense	A	12	White-orange	Orange	+	−	+	+	w	4
2 H1	IHEM 25437	Belgium	Tinea capitis	T. soudanense	A	10	Orange	Orange	+	−	+	w	−	−
2 H1	IHEM 25438	Belgium	Skin	T. soudanense	A	13	Orange	Orange	+	−	+	w	−	−
2 H1	IHEM 26433	Belgium	Tinea capitis	T. soudanense	C	13	Yellow-orange	Orange	+	−	+	+	−	−
2 H1	IHEM 26434	Belgium	Tinea capitis	T. soudanense	A	13	Yellow-orange	Orange	+	−	+	+	−	5
2 H2	IHEM 26699	Belgium	Skin	T. soudanense	A	15	White	Orange	+	−	+	+	−	−
2 H1	CBS 384.89	Sweden	Nail	T. soudanense	A	9	Yellow	Orange	+	−	+	+	+	−
2 H2	IHEM 26026	Belgium	Tinea capitis	T. soudanense	A	13	Yellow-orange	Orange	+	−	+	+	−	−
2 H2	CBS 453.59	Chad	/	T. soudanense	A	21	White	Orange	−	−	+	+	+	−
2 H2	CBS 360.62	Togo	Nail	T. soudanense	A	12	White	Light brown	−	−	−	+	+	3
2 H2	IHEM 22465	Belgium	Tinea capitis	T. soudanense	A	14	Yellow-orange	Orange	+	−	+	+	w	−
2 H2	CBS 452.61	Zaire	/	T. violaceum	A	15	White	Orange	+	−	−	+	+	−
2 H2	CBS 303.38	/	/	T. soudanense	C	28	White	Orange	+	−	−	+	w	−
2 H2	IHEM 22798	Belgium	Tinea capitis	T. soudanense	A	12	Yellow	Orange	+	−	+	+	−	−
														
3 H5	CBS 809.69	Germany	/	T. rubrum	A	20	White	White-brown	+	−	−	+	++	−
3 H5	CBS 289.86	Canada	Buttock	T. rubrum	C	18	White	Brown	+	+	−	+	+	−
3 H5	CBS 127447	Turkey	Scalp	T. soudanense	A	20	White	White-brown	+	−	−	+	w	−
3 H5	CBS 131555	Thailand	/	T. soudanense	A	14	White	Brown	+	−	−	+	+	−
3 H5	IHEM 2770	Belgium	Foot	T. rubrum	C	20	White	Yellow	+	−	−	+	+	−
3 H5	HS 214-8900	China	Skin	T. soudanense	C	20	White	Yellow-brown	+	−	−	+	+	−
3 H5	HS 215-960	China	Ear	T. soudanense	C	22	White	White-brown	+	+	−	+	w	−
3 H5	HS 97-146	China	/	T. rubrum	A	23	White	Yellow-brown	+	−	−	+	+	−
3 H5	HS 214-2545	China	/	T. soudanense	C	25	White	Yellow-brown	+	−	−	+	w	2
3 H5	CBS 376.49^T^	Congo	Tinea cruris	T. soudanense	C	17	White	Cream	+	−	−	+	w	−
3 H5	CBS 392.58 NT	Netherlands	Foot	T. rubrum	A	17	Cream	Cream	+	−	−	+	+	10
3 H5	CBS 189.69	Netherlands	Nail	T. rubrum	A	18	White	Orange-brown	+	−	−	+	++	−
3 H5	CBS 807.69	Germany	/	T. soudanense	C	13	White	Light brown	−	−	−	+	w	−
3 H5	CBS 100081^T^	Canada	Contaminant	*/*	C	26	White	White-yellow	+	−	−	+	++	8
3 H5	CBS 100084^T^	Canada	Skin	T. soudanense	C	18	White	Cream-brown	+	−	−	+	++	3
3 H5	CBS 115316	Greece	Skin	T. rubrum	A	19	White	Yellow	+	−	−	+	w	−
3 H5	CBS 115317	Bulgaria	/	T. rubrum	C	19	White	Brown	+	−	−	+	+	−
3 H5	CBS 116716	Turkey	Skin	T. soudanense	A	16	White	Yellow-brown	+	−	−	w	w	8
3 H5	CBS 117539	Greece	Nail	T. rubrum	A	17	White	Orange	+	−	−	w	+	−
3 H5	CBS 118892	Germany	Nail	T. soudanense	A	21	White	Yellow	+	−	−	+	w	−
3 H5	CBS 130927	Iran	Foot	*/*	A	18	White	Brown	+	−	−	+	w	−
3 H5	CBS 131904	Thailand	/	T. rubrum	A	15	White	Brown	+	−	−	+	+	−
3 H5	CBS 131906	Thailand	/	*/*	C	19	White	Brown	+	−	−	+	+	−
3 H5	CBS 139496	India	/	*/*	C	22	White	Yellow	+	−	−	+	w	−
3 H5	IHEM 13801	Mozambique	Skin	T. rubrum	A or B	19	White	Cream-brown	+	−	−	+	w	−
3 H5	IHEM 13809	Guiana	Skin	T. rubrum	A or B	22	White	Cream	−	−	−	+	+	−
3 H5	IHEM 25807	Italy	Tinea capitis	T. rubrum	C	21	White	Yellow-brown	+	−	−	+	+	−
3 H5	IHEM 26594	Belgium	Skin	T. rubrum	C	28	White	Yellow-brown	+	+	−	+	+	−
3 H5	HS 217-546	China	Nail	T. soudanense	C	13	White	Brown	+	−	−	+	w	−
3 H5	HS 217-4895	China	Nail	T. soudanense	C	11	White	Yellow-brown	+	−	−	+	w	−
3 H5	HS 217-5305	China	Face	T. soudanense	C	4	White	Orange	−	−	−	+	+	2
3 H5	HS 217-5388	China	Foot	T. soudanense	C	20	White	Yellow	−	−	−	+	w	−
3 H5	HS 214-5151	China	Face	T. rubrum	C	21	White	Yellow	−	−	−	+	+	−
3 H5	HS 214-5152	China	Foot	T. rubrum	A	21	White	Yellow-brown	+	−	−	+	w	−
3 H5	HS 215-28581	China	Foot	T. soudanense	A or B	23	White	Yellow-brown	+	−	−	+	+	−
3 H5	HS 215-28582	China	Face	T. soudanense	C	16	White	Brown	+	−	−	+	+	3
3 H5	HS 215-8195	China	Face	*/*	C	10	White	Cream	+	−	−	+	w	−
3 H5	HS 217-68	China	Scrotum	T. rubrum	C	16	White	Yellow-brown	+	−	−	+	w	−
3 H5	HS 215-528	China	Scalp	T. soudanense	/	18	White	White-brown	+	+	−	+	w	−
3 H5	HS 215-898	China	Skin	T. rubrum	C	17	White	Cream	+	−	−	+	+	−
3 H5	HS 215-7047	China	Skin	T. soudanense	C	14	White	Brown	+	−	−	+	−	3
3 H5	HS 214-9316	China	Skin	T. soudanense	A	22	White	Yellow	+	−	−	+	+	−
3 H5	HS 215-2607	China	Face	T. soudanense	C	22	White	Yellow-brown	+	−	−	+	w	−
3 H5	HS 215-8620	China	/	T. soudanense	C	16	White	Yellow	+	−	−	+	+	−
3 H5	HS 216-1070	China	/	*/*	C	18	White	White-brown	+	−	−	+	+	−
3 H5	HS 317-121	China	/	T. soudanense	C	14	White	Yellow-brown	+	−	−	+	+	−
3 H5	CBS 288.86	Canada	Contaminant	T. rubrum	C	17	White	Yellow	+	−	−	+	+	−
3 H5	HS 214-913	China	Face	T. rubrum	C	22	White	Yellow	−	−	−	+	+	−
3 H5	HS 217-69	China	Foot	T. rubrum	C	18	White	Yellow-brown	+	−	−	+	+	3
3 H5	HS 217-70	China	Nail	T. rubrum	C	18	White	Yellow-brown	+	−	−	+	+	−
3 H5	CBS 202.88	Canada	Foot	*/*	C	19	White	Yellow-brown	+	−	−	+	w	−
3 H6	CBS 304.60	/	/	T. rubrum	A	18	White	Yellow	+	+	−	+	+	−
3 H6	CBS 191.69	Cameroon	/	T. rubrum	A	15	White	Brown	+	+	−	+	+	−
3 H6	CBS 102856	Italy	Nail	T. rubrum	A	18	White	Brown	+	−	−	+	+	−
3 H6	HS 217-5111	China	Nail	T. soudanense	C	21	White	Yellow-brown	+	−	−	+	+	−
3 H6	CBS 287.86	Canada	Skin	T. rubrum	A	18	White	Brown	−	+	−	+	++	−
3 H6	CBS 592.68^T^	Guinea	Skin	T. soudanense	A	18	White	Yellow	+	−	−	+	+	−
														
4 H4	CBS 417.52	Switzerland	/	T. rubrum	A	24	White	White-brown	+	+	−	+	−	−
4 H4	CBS 734.88	Spain	Chin	*/*	C	21	White	Yellow-brown	+	−	−	+	−	−
4 H4	CBS 735.88	Spain	Chin	*/*	A	20	White	Yellow-brown	+	−	−	+	−	−
5 H3	CBS 517.63^T^	Burundi	Tinea capitis	T. soudanense	A	25	White	White	+	−	−	+	−	−
5 H3	CBS 422.67	Zaire	/	T. rubrum	A	15	White	Yellow	+	−	−	+	w	−
5 H3	CBS 518.63	/	/	T. violaceum	C	24	White	Cream	+	−	−	+	−	−

a ITS, ITS number according to the phylogenetic analysis and haplotype; Geography, the geographical source of the isolates; Source, clinical information for the strains (E, environmental); MALDI-TOF MS, matrix-assisted laser desorption ionization–time of flight mass spectrometry; AFLP, amplified fragment length polymorphism (m, data missing); D, average diameter (in millimeters) of the colony at 2 weeks; Obverse, color of the front side of colony at 2 weeks; Reverse, color of the reverse side of the colony at 2 weeks; Mi, microconidia; Ma, macroconidia; Rb, reflexive branches; Ur, urea hydrolysis test result; Ke, keratin azure test result; Tw, Tween 80 opacity test results at 2 weeks (the value indicates positive results and the difference between the diameter [in millimeters] of the colony and the halo); +, positive; −, negative; w, weak; /, no data. NT, neotype.

### Phenotypes.

**(i) Morphology.** Isolates were grown on Sabouraud’s glucose agar (SGA; Difco) in 90-mm culture plates at 24°C. Colony diameters and the colors of the obverse and reverse sides were recorded after 2 weeks. Strains grown on malt extract agar (MEA; Oxoid) were examined by microscopy, and the presence of micro- and macroconidia and reflexive branches was recorded.

**(ii) Urea hydrolysis.** Strains were grown on Christensen’s urea agar (Oxoid) slants at 24°C for 1 week. A medium color change from orange to purple/red or pale pink indicated the strong or weak secretion of urease, respectively.

**(iii) Hair perforation.** Strains HS 217-5329, CBS 555.84, IHEM 4887, CBS 131555, HS 214-5151, IHEM 2770, IHEM 1284, CBS 360.62, IHEM 13459, and IHEM 4711 were randomly selected for use in the hair perforation test. Blond children’s hairs were inoculated with fungi suspended in sterilized H_2_O and examined microscopically after 4 weeks of incubation. Nannizzia gypsea CBS 130813 was used as a positive control. Marked localized areas of pitting suggested the ability to perforate hair.

**(iv) Keratinase.** Keratin azure agar was prepared as described by Scott and Untereiner (16) and dispensed horizontally in sterilized test tubes. First, 5 ml basal medium (2.5% agar, 0.05% MgSO_4_·7H_2_O, 0.05% KCl, 0.05% K_2_HPO_4_, 0.01% ZnSO_4_·7H_2_O, 0.01% FeSO_4_·7H_2_O, 0.003% CuSO_4_) was applied in each tube; after solidification, 0.5 ml upper layer (1% agar, 0.05% MgSO_4_·7H_2_O, 0.05% KCl, 0.05% K_2_HPO_4_, 0.01% ZnSO_4_·7H_2_O, 0.01% FeSO_4_·7H_2_O, 0.003% CuSO_4_, 4 mg/ml keratin azure) was applied. Mycelia grown on malt extract agar (MEA; Oxoid) were inoculated and incubated at 37°C for 1 month. The appearance of a blue color in the basal layer indicated keratin degradation.

**(v) Tween 80 opacity test.** Tween 80 agar medium was prepared with 10.0 g Bacto peptone, 5.0 g NaCl, 0.1 g CaCl_2_, and 15.0 g agar per 1,000 ml distilled water (17). After autoclaving, the medium was cooled to about 50°C and 5 ml of autoclaved Tween 80 was added. Culture plates (90 mm) were filled with 25 ml medium, inoculated, and incubated at 24°C for 2 weeks. Fungal growth was examined weekly; the presence of a halo due to calcium precipitation around the colony indicated lipolytic activity.

### Molecular methods.

**(i) DNA extraction.** Fungal material was transferred to 2-ml screw-cap tubes filled with 490 μl 2× cetyltrimethylammonium bromide (CTAB) buffer (2% CTAB, 100 mM Tris-HCl, 20 mM EDTA, 1.4 M NaCl) and 6 to 10 acid-washed glass beads. Then, 10 μl of proteinase K was added and the components were mixed thoroughly on a MoBio vortex mixer (MoBio Laboratories, Carlsbad, CA, USA) for 10 min. After incubation for 60 min at 60°C, 500 μl chloroform-isoamyl alcohol (24:1) was added and the mixture was shaken for 2 min; the tubes were centrifuged for 10 min at 14,000 rpm, and the supernatants were collected into new Eppendorf vials. To 400 μl of the DNA sample, 2/3 vol (270 μl) ice-cold isopropanol was added, the mixture was centrifuged again at 14,000 rpm for 10 min, and the upper layer was dissolved in 1 ml ice-cold 70% ethanol. The tubes were centrifuged again at 14,000 rpm for 2 min, air dried, and resuspended in 50 μl TE (Tris-EDTA) buffer. Samples were stored at −20°C until analysis.

**(ii) ITS PCR.** Primers ITS1 ( TCCGTAGGTGAACCTGCGG) and ITS4 ( TCCTCCGCTTATTGATATGC) were applied for amplification, and 10 to 100 ng DNA was added to 25 μl PCR buffer. The PCR program was as follows: 95°C for 4 min, followed by 35 cycles of 95°C for 45 s, 52°C for 30 s, and 72°C for 2 min, with a delay at 72°C for 7 min. The PCR products were visualized on 1% agarose gels and sequenced using an ABI BigDye Terminator (v3.1) cycle sequencing kit. The sequencing reactions were done at 95°C for 1 min and 30 cycles of 95°C for 10 s, 50°C for 5 s, and 60°C for 4 min on an ABI 3730XL automatic sequencer (Applied Biosystems, Foster City, CA, USA) with the ABI Prism BigDye Terminator cycle sequencing kit. The sequences were edited and assembled with the SeqMan program (DNAStar, Madison, WI, USA), manually corrected, and aligned using the MAFFT server (www.ebi.ac.uk/Tools/msa/mafft/) with default parameters. A phylogenetic tree was generated using the maximum likelihood method with MEGA (v7.0) software based on the general time-reversible model. T. erinacei IHEM 19618 and T. verrucosum IHEM 5480 were used as outgroups. The sequences of newly sequenced strains were deposited in GenBank; the accession numbers are listed below. The accession numbers of all the strains used in this study are listed in Table S1 in the supplemental material.

**(iii) ITS diversity.** Polymorphisms in ITS sequences of 175 strains from the T. rubrum complex were detected using DNASP (v5.10) software. The number of polymorphic sites (*S*), haplotype diversity (Hd), and the average number of pairwise nucleotide differences per site (π) were calculated (gaps/missing data were included). A haplotype network was constructed in the Network (v4.6.1.0) program (Fluxus-Technology, UK) using the median-joining method.

**(iv) AFLP.** AFLP genotyping was performed as previously described (18). Selective primers HpyCH4IV-C (50-fluorophore-GTAGACTGCGTACCCGTC-30) and MseI-TGAG (50-GATGAGTCCTGACTAATGAT-30) were used in the restriction ligation procedure. Fragments were analyzed on an ABI3500xL genetic analyzer (Applied Biosystems, UK); for this purpose, the amplicons were 10× diluted with double-distilled H_2_O (ddH_2_O), and 1 μl of this dilution was added to 0.1 μl the LIZ600 internal size marker (Promega, Leiden, The Netherlands) and 8.9 μl ddH_2_O. Raw data were further processed by using BioNumerics (v7.5) software (Applied Maths, Sint-Martens Latem, Belgium), and a dendrogram was generated based on the unweighted pair group method using average linkages (UPGMA) algorithm.

**(v) MALDI-TOF MS.** MALDI-TOF MS was performed as described by Packeu et al. (19). Briefly, a reference spectrum database was constructed based on the method of Cassagne et al. (20) and Normand et al. (21) and included reference strains representing all species from the T. rubrum complex available in the BCCM/IHEM collection (Table S2).

The isolates were incubated for 7 days on SGA with chloramphenicol. Some slow-growing strains were tested after 12 to 14 days. Protein was extracted with formic acid-acetonitrile. Reference metaspectra were established based on the mass spectra from four subcultures of the same strain. Samples were analyzed in quadruplicate, i.e., with four parallel analyses of the same extract. Spectra were recorded in the positive linear mode in a mass range of from 2 to 20 kDa using MALDI Biotyper automation control software (Bruker Daltonics, Bremen, Germany). The spectra of each spot were compared with those in the reference library and analyzed with MALDI Biotyper (v3.0) software (Bruker), with log scores (LS) ranging from 0 (no match) to 3 (perfect match). The MS-based identification was considered reliable only if at least three out of the four spots resulted in the same identification with an LS of ≥1.70. The spectra are maintained in a database at Sciensano and are available upon request.

### Data availability.

The ITS sequences of newly sequenced strains were deposited in GenBank under accession numbers MK806589 to MK806666.

## RESULTS

### ITS diversity.

**FIG 1:**
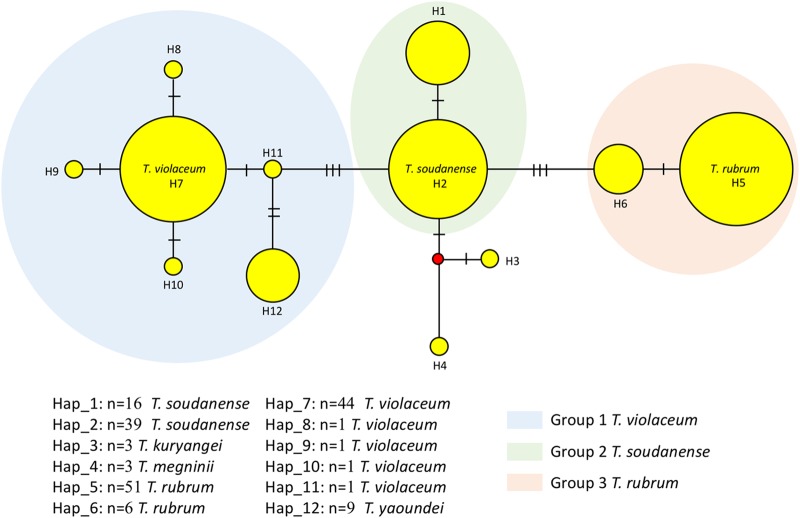
Medium-joining haplotype network of the T. rubrum complex generated from the ITS sequences of 175 strains. Yellow circles represent haplotypes; the circle size indicates frequency. Haplotypes were grouped as identified by ITS data. Mutational steps are indicted by hashtags; red dots (median vectors) represent hypothetical haplotypes.

ITS alignment was performed for 175 strains of the T. rubrum complex; strains T. erinacei IHEM 19618 and T. verrucosum IHEM 5480 were added as an outgroup in the phylogenetic analysis. The total alignment length (with gaps) was 565 bp; the number of invariable sites was 514, and the number of variable sites was 51, among which 40 were gaps/missing data (singleton variable sites, 2; parsimony-informative sites, 9). The results of ITS haplotype analysis are shown in Fig. 1. In total, 12 haplotype patterns were revealed (Hd, 0.7938).

A phylogenetic tree based on the ITS sequences was constructed for 175 strains using the maximum likelihood method (see Fig. S1 in the supplemental material). Three main groups differing by a few single nucleotide polymorphisms (SNPs) were revealed in both haplotype analysis and the phylogenetic tree. Group 1 (haplotype 7 [H7] to H12 in the haplotype network, 57 strains) contained the neotype of T. violaceum, CBS 374.92. Within this group, nine strains (H12), including the type strain T. yaoundei CBS 305.60, deviated in one SNP (T-C) at position 217 and are referred to as group 1A. CBS 141826 (H8) and CBS 141829 (H9) were found to have mutations at position 375 (A-G) and 525 (C-T), respectively. An insertion of C was found at position 117 in IHEM 13375 (H10), and a deletion of A was found at position 236 in CBS 118535 (H11). These indels and SNPs led to small deviations in the tree caused by strains with different haplotypes.

Group 2 contained 39 identical strains (H2), including T. soudanense neotype strain IHEM 19751; among this group, 16 strains (H1) deviated by a deletion of 37 bases. Group 3 was observed at a distance of 4 bp from group 2; it contained 57 isolates, including 6 strains differing by 1 SNP, which were referred to as group 3A (H6).

Six strains, including T. kuryangei type strain CBS 517.63 and three T. megninii strains, could not be clearly assigned to any of the three groups and were considered to have an ambiguous affiliation (H3, H4); they were provisionally excluded from the study. Based on the positions of the (neo)type strains in groups 1 to 3, we conclude that group 1 represents T. violaceum (with group 1A being a variant), group 2 represents T. soudanense, and group 3 represents T. rubrum. The T. violaceum group contained T. violaceum var*. indicum* and T. glabrum, with T. yaoundei slightly deviating as group 1A. The T. soudanense group included T. circonvolutum, T. gourvilii var. *intermedium*, and T. gourvilii, and the T. rubrum group contained type strains of T. fischeri, T. flavum, T. fluviomuniense, T. kanei, T. pedis, T. raubitscheckii, and T. rodhainii. The type strain of T. kuryangei took an uncertain position somewhat outside of groups 1 to 3.

### Phenotype.

The strains in group 1/1A (T. violaceum*/*T. yaoundei) had an average growth rate of 12.98 ± 4.31 mm/2 weeks. After 2 weeks, T. violaceum colonies were invariably glabrous, leathery, and wrinkled and had a white, cream, or yellow color (occasionally with brownish or reddish tinges) at the obverse and a white, cream, or brown color (occasionally with reddish tinges) at the reverse; the colonies of some strains acquired a darker, red-brown or dark purple color (Table 1). Group 2 (T. soudanense) strains had an average growth rate of 14.05 ± 3.69 mm/2 weeks, and the colonies had a similar texture, with the colonies also being wrinkled and usually showing a white or yellow to orange color at the obverse and an orange to orange-yellow color (occasionally with a brownish tinge) at the reverse. Group 3 (T. rubrum) strains had an average growth rate of 18.33 ± 3.94 mm/2 weeks and formed fluffy colonies with a white (occasionally cream) color at the obverse and a white, cream, yellow, or brown (occasionally orange) color at the reverse.

Only three strains in group 1/1A (5.26%) showed reflexive branching, while almost all strains in group 2 (87.27%) showed this characteristic. Reflexive branching was strictly absent from group 3. Microconidia were detected in 15.79%, 90.91%, and 87.72% strains in group 1, group 2, and group 3, respectively, whereas macroconidia were observed exclusively in group 3 (12.28% strains).

### Physiology.

**FIG 2:**
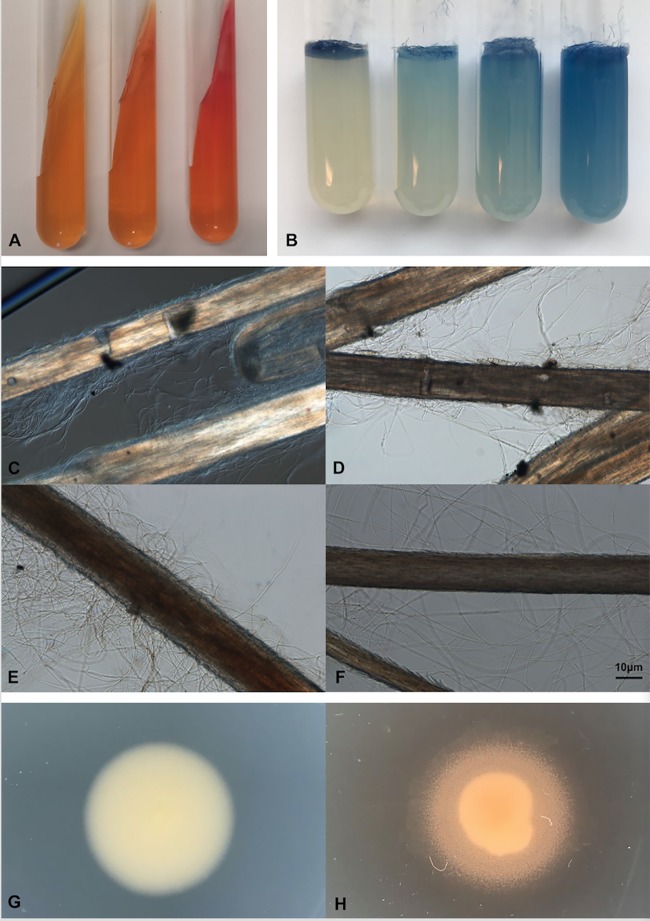
Phenotypic methodology. (A) Christensen’s urea slants. Negative-control (left), weak (middle), and positive (right) results are shown. (B) Keratin azure agar. From left to right, negative (score, 1), weak (score, 2), positive (score, 3), and strongly positive (score, 4) results are shown. (C to F) Hair perforation by the positive control, Nannizzia gypsea CBS 130813 (C, D), Trichophyton rubrum (E), and Trichophyton soudanense (F). (G, H) Tween 80 opacity; (G) negative result for T. rubrum; (H) positive result for T. violaceum.

The physiological characteristics of each strain are shown in Table 1. Urea hydrolysis was observed in all strains; representative negative, weak, and positive results are shown in Fig. 2A. Weak urea hydrolysis (pale pink medium) was observed in 8.77%, 21.81%, and 3.51% of the strains in group 1, group 2, and group 3, respectively.

Almost all group 3 strains (with one exception) showed the ability (minimally weak to excellent) to degrade and assimilate keratin, whereas 54.39% and 52.73% of the strains in groups 1 and 2, respectively, failed to hydrolyze keratin. There was no statistically significant difference in keratin degradation between groups 1 and 2 (*P* = 0.86), whereas their difference with group 3 was significant (*P* < 0.001) (Fig. 2B).

Among randomly selected strains incubated with blond children’s hair, none produced perforations (Fig. 2E and F); at most, some hyphae were found to be attached to the hair surface (Fig. 2E), whereas Nannizzia gypsea CBS 130813, used as a positive control, showed localized areas of pitting (Fig. 2C and D).

The Tween 80 opacity test was used to examine the lipolytic ability of the analyzed dermatophytes. Most group 1 strains (47 out of 57, 82.46%) showed a positive response, manifested by a large halo of precipitate around the colonies (7.89 mm, on average, after 2 weeks of incubation) (Fig. 2H). In contrast, most strains in groups 2 and 3 had no or weak lipolytic activity; only a few of them showed lipolysis (9/57 and 13/55, respectively; average halo, 4.67 and 4.62 mm, respectively) (Fig. 2G). There was no difference between groups 2 and 3 (*P* = 0.30), but their difference with group 1 was statistically significant (*P* < 0.001).

### MALDI-TOF MS.

A reference database including the main spectrum profiles (MSPs) from T. rubrum (*n* = 6), T. kuryangei (*n* = 1), T. soudanense (*n* = 21), T. violaceum (*n* = 9), and T. yaoundei (*n* = 3) was established (Table S2); 81.48% of strains in group 1 and 96.15% in group 2 were identified as T. violaceum and T. soudanense, respectively. All strains in ITS group 1A were identified as T. yaoundei. However, only 41.18% of strains in group 3 and 66.67% of strains in group 3A showed an agreement with the ITS data. Among the misidentified strains in group 3/3A, 24 (42.11%) were classified as T. soudanense.

### AFLP.

**FIG 3:**
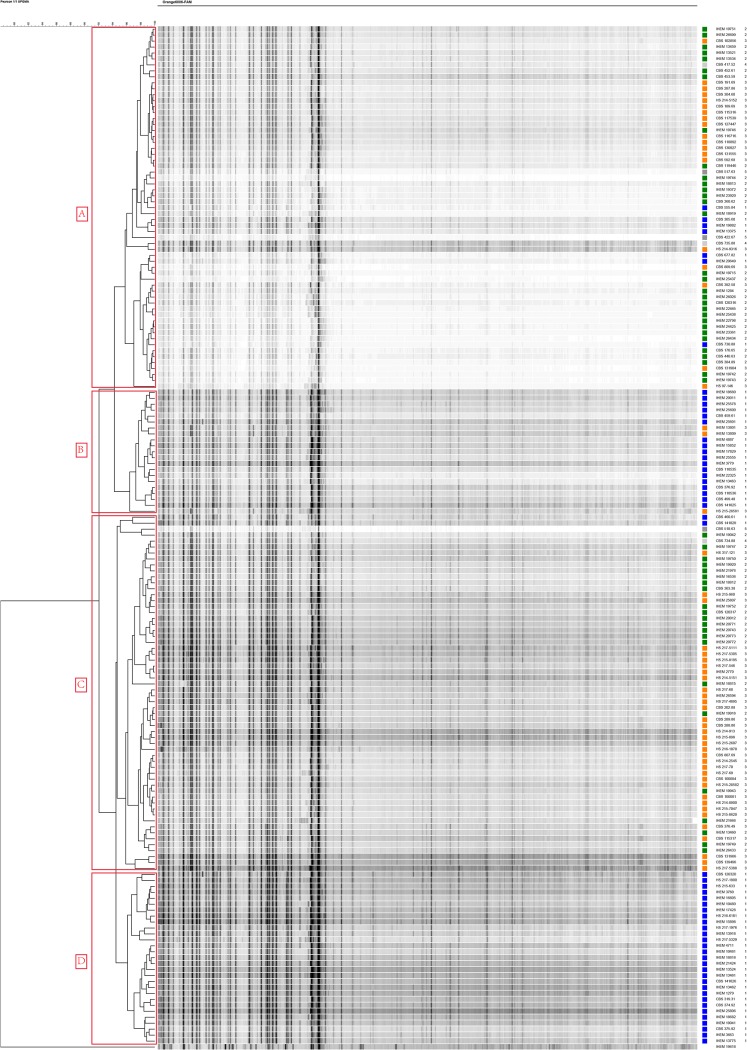
UPGMA-derived dendrogram of AFLP fingerprints of 172 isolates, with T. erinacei IHEM 19618 being the outgroup. Similarities in percentages are indicated with the scale bar in the upper left corner. Strain numbers and ITS groups are listed. FAM, 6-carboxyfluorescein.

A total of 172 strains were typed to obtain an AFLP dendrogram. As was found with the ITS data, strains showed a high similarity in their AFLP fingerprint profiles. Although four patterns (AFLP-A to AFLP-D) were distinguishable in the dendrogram based on an arbitrary cutoff value of ≤95% similarity, the difference between patterns was not significant. Group 1 comprised strains with all four patterns (A, 12.50%; B, 32.14%; C, 3.57%; D, 51.79%), whereas group 2 strains had two patterns (A, 58.49%; C, 41.51%) and group 3 strains had three patterns (A, 33.93%; B, 5.36%; C, 60.71%). Most strains in groups 2 and 3 had patterns A and C, and group 1 strains mainly showed patterns B and D. Pattern D was detected exclusively in group 1. T. kuryangei and T. megninii strains had only patterns A and C (Fig. 3).

## DISCUSSION

Taxonomic entities among anthropophilic dermatophytes were shown to be similar in their molecular characteristics (6, 22); however, our present data show the existence of three species in the Trichophyton rubrum complex, judging from the ITS rDNA data. Although none of the physiological, morphological, geographical, MALDI-TOF MS, or AFLP data were unambiguously diagnostic, we revealed different trends that were statistically significant. Groups 1, 2, and 3 contained the type strains of Trichophyton violaceum, T. soudanense, and T. rubrum, respectively; being the oldest epithets in these clusters, they provide correct names for the three groups. Group 1 contained a slightly deviating group 1A around the type strain of T. yaoundei from the Congo, but more data are required to establish whether it can be considered a separate taxon. Group 3 contained type strains of T. fischeri, T. fluviomuniense, T. kanei, T. raubitschekii, and T. rodhainii, all of which, consequently, can be regarded as proven synonyms of T. rubrum. T. violaceum and T. soudanense are prevalently found on the scalp (80.85% and 71.43% of strains from human sources, respectively), whereas T. rubrum is mostly found on glabrous skin (6.98% of strains from human sources). The geographical origin of the strains is somewhat difficult to trace back because of the increased traveling and migration of humans. Disregarding the isolates from Western countries, it was found that T. rubrum and T. violaceum have a global distribution, whereas T. soudanense is limited to Africa. Cases of T. soudanense infection reported in the United States were also observed among patients of African descent (23).

Colony appearance has been classically used to distinguish species in the T. rubrum complex, and our results confirmed previous data. Colonies of T. violaceum and T. soudanense are glabrous and grow slower than those of T. rubrum; most isolates of T. violaceum lack microconidia, which are generally present in T. soudanense and T. rubrum. Rather unexpectedly, reflexive branching appeared to be a diagnostic marker, as it is very common for T. soudanense and rarely or never observed in the other two species. Macroconidia occur only in T. rubrum, but as they are easily lost after repeated subculturing, this phenotypic trait has limited value. In the reverse, most colonies of T. violaceum are colored cream, those of T. soudanense are yellow-orange, and those of T. rubrum had brown tinges. The colony coloration observed in the present study was obviously influenced by prolonged culturing, which usually leads to the loss of pigmentation; nevertheless, a trend was detected. A naphthaquinone derivative, xanthomegnin, the main pigment synthesized by the members of the T. rubrum complex, was first isolated from a strain identified as T. megninii and later from the other strains of the complex (24–26); it could also be detected in human skin and nails colonized by T. rubrum (27). The pH-reversible naphthaquinone pigment xanthomegnin is the main pigment responsible for the observed colony colors. The darker tinges in T. rubrum are possibly associated with higher metabolic activity, leading to higher ammonium production and alkaline pH.

Almost all analyzed strains were positive for urea hydrolysis at 24°C, indicating urease expression. Urea broth and agar have been reported to be useless for species identification within the T. rubrum complex, although T. rubrum tends to hydrolyze urea slower than T. mentagrophytes (28). Urease activity should no longer be considered a criterion for differentiation of T. rubrum var. *raubitschekii*, as both taxa showed positive results (29).

Hydrolysis of Tween 80, derived from polyethoxylated sorbitan, and oleic acid is used as an indicator of the production of lipolytic enzymes (17). Lipases might be associated with different types of hair invasion of dermatophytes. Trichophyton violaceum and T. soudanense are mostly involved in tinea capitis; clinical forms of superficial infections vary from asymptomatic carriage to kerion, favus, scalp penetration, and black dot infection (30–32). Glabrous skin and the scalp differ in hair size and density and the abundance of sebaceous glands secreting oily material into hair follicles (33). According to the Tween 80 opacity test, T. violaceum had a higher lipolytic ability than the other species. We might speculate that T. rubrum on glabrous skin is directly involved in degradation of the epidermis of the skin, whereas T. violaceum and T. soudanense grow into the hair follicle through the sebaceous gland, reaching the medulla entering the more lipid-rich central hair shaft. This hypothesis is consistent with the fact that the prevalent hair infection type caused by the latter species is endothrix, resulting in the short, broken hairs clinically observed in tinea capitis (34, 35). The reduced Tween 80 hydrolysis in T. soudanense may be associated with a drier hair type prevalent in Africa; however, this speculation requires further experimental confirmation. T. violaceum has been thought to affect children more frequently than adults, because of the possible fungistatic activity of long-chain fatty acids in sebum secreted by sebaceous glands, whose activity increases with age (36). In addition, the higher incidence of tinea capitis in children might be also linked to underdeveloped immunity. The dual function of sebum in fungal infections should be further investigated.

Microscopy examination of T. violaceum-infected hair reveals endothrix accompanied by multiple fungal spores inside the hair, although this species is usually nonsporulating *in vitro*. The hair perforation test was consistently negative for all analyzed strains, confirming earlier findings (28, 37) and indicating that fungi are incapable of degradation of the keratinous hair cuticle to reach the softer cortex *in vitro*. T. rubrum showed the highest keratin azure degradation, which was either absent or weak in the other species. The keratin azure test was first performed in fungi by Scott and Untereiner (16), who reported that T. rubrum had weak dye release after 6 weeks. Currently, keratin azure is widely used as a substrate to reveal keratinase activity (38). We modified the protocol by applying the agar into normal tubes instead of square bottles for better observation of the results for these slow-growing fungi. Most T. rubrum strains exhibited blue dye release after 1 month of incubation, and almost half of T. violaceum strains and one-third of T. soudanense strains showed some ability to degrade keratin. The nonspecific serine proteases subtilisin 3 (Sub3) and Sub4, detected in T. rubrum culture supernatants (39), were confirmed to degrade keratin azure, and both were predicted to be expressed in T. rubrum and T. violaceum by whole-genome analysis (40). Expression of keratinases, such as Sub3 and Sub4, and concomitant keratin degradation appear to be rather variable within a single species.

MALDI-TOF MS could separate most T. violaceum strains from T. rubrum and T. soudanense but had an insufficient discriminatory power to unambiguously discriminate between T. rubrum and T. soudanense. A recent study on dermatophyte identification using MALDI-TOF MS suggested that inclusion of T. soudanense in the database potentially leads to the misidentification of T. rubrum (41). Besides, some strains failed to be identified with MALDI-TOF MS because of poor growth on agar plates with an extended culturing time.

AFLP genotyping revealed a high degree of similarity among groups 1 to 3, indicating their close genetic relationship. Notably, much larger differences were found using the same amplification system between species of *Sporothrix* (42) and *Cryptococcus* (43), which, until recently, were considered species complexes, and even within the single species Hortaea werneckii (44). Approximate AFLP groups A to D were distinguished on the basis of the total profiles sorted by UPGMA clustering of the total profiles. Trichophyton violaceum (ITS group 1) contained strains divided over all AFLP patterns, A to D, confirming an ancestral position, as was noted with the ITS data (Fig. 1), to T. rubrum and T. soudanense, which shared exclusively profiles A and C. On the basis of the total profiles, including minor bands, three strains clustered in AFLP group B, but on the basis of major bands only, IHEM 13801, IHEM 13801, and HS 215-28581 would more appropriately be classified in group A. The derived characteristics of T. rubrum may explain the above-described poor performance of MALDI-TOF MS, with T. rubrum showing 43.9% mismatches, while T. soudanense and T. violaceum were recognized nearly correctly.

In conclusion, T. violaceum, T. soudanense, and T. rubrum show coherent differences in independent parameters of clinical features, morphology, physiology, and genetics, although none of these parameters is strictly diagnostic. Genetically, the entities are very similar, suggesting a very short time of evolution. Combined with phenotypic differences and clinical predilection, in the absence of sexuality, we conclude that these are separate entities in sympatric evolution but with incomplete lineage sorting, as has also been observed in other recently evolving dermatophytes (45). In view of the clinical significance of these fungi, which has long been recognized in dermatology, we recommend maintaining the entities at the species level. The species, with a confidence level of >90%, cause different types of infection and show distinct colony morphology and microscopic features. Physiological tests on keratin degradation and lipolysis indicate differences in ecological specialization but are not practical as identification criteria, and AFLP and MALDI-TOF MS do not have sufficient discriminatory power to distinguish between all species reliably. Clinical manifestations remain a primary criterion for identification, which should be best confirmed by ITS sequencing. For laboratories lacking access to ITS sequencing capabilities, clinical manifestations could be combined with morphological characteristics of the strains for identification. T. violaceum and T. soudanense are mostly involved in tinea capitis. The distance in the ITS barcoding gene between T. violaceum and T. rubrum is 6 bp (1.06%), which is beyond the generally applied limit to be accepted as different species. Genetic differences with T. soudanense are smaller, but its endemism in northern Africa as a cause of tinea capitis, supplemented with its phenotypic differences, makes distinction of this entity clinically meaningful.

## Supplementary Material

Supplemental file 1

Supplemental file 2

Supplemental file 3
