# Supplementary material for: Species Distinction in the Trichophyton rubrum Complex
Source: J Clin Microbiol. 2019 Aug 26;57(9):e00352-19. doi: 10.1128/JCM.00352-19 (PMC6711931; doi:10.1128/JCM.00352-19)
Supplement: Supplemental file 1 [file JCM.00352-19-s0001.pdf]

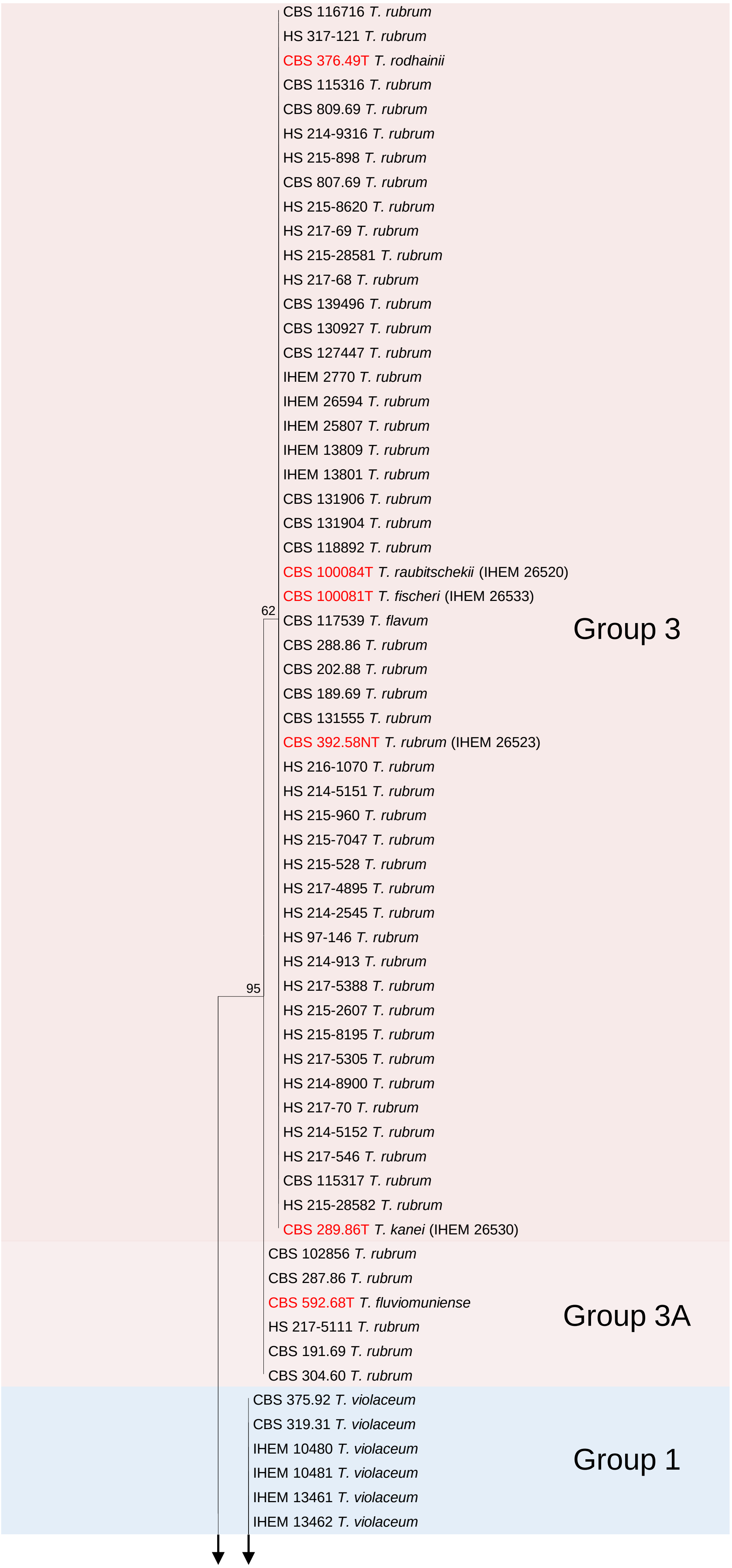
CBS 116716 *T. rubrum*
HS 317-121 *T. rubrum*
CBS 376.49T *T. rodhainii*
CBS 115316 *T. rubrum*
CBS 809.69 *T. rubrum*
HS 214-9316 *T. rubrum*
HS 215-898 *T. rubrum*
CBS 807.69 *T. rubrum*
HS 215-8620 *T. rubrum*
HS 217-69 *T. rubrum*
HS 215-28581 *T. rubrum*
HS 217-68 *T. rubrum*
CBS 139496 *T. rubrum*
CBS 130927 *T. rubrum*
CBS 127447 *T. rubrum*
IHEM 2770 *T. rubrum*
IHEM 26594 *T. rubrum*
IHEM 25807 *T. rubrum*
IHEM 13809 *T. rubrum*
IHEM 13801 *T. rubrum*
CBS 131906 *T. rubrum*
CBS 131904 *T. rubrum*
CBS 118892 *T. rubrum*
CBS 100084T *T. raubitschekii* (IHEM 26520)
CBS 100081T *T. fischeri* (IHEM 26533)
62
CBS 117539 *T. flavum*
Group 3
CBS 288.86 *T. rubrum*
CBS 202.88 *T. rubrum*
CBS 189.69 *T. rubrum*
CBS 131555 *T. rubrum*
CBS 392.58NT *T. rubrum* (IHEM 26523)
HS 216-1070 *T. rubrum*
HS 214-5151 *T. rubrum*
HS 215-960 *T. rubrum*
HS 215-7047 *T. rubrum*
HS 215-528 *T. rubrum*
HS 217-4895 *T. rubrum*
HS 214-2545 *T. rubrum*
HS 97-146 *T. rubrum*
HS 214-913 *T. rubrum*
95
HS 217-5388 *T. rubrum*
HS 215-2607 *T. rubrum*
HS 215-8195 *T. rubrum*
HS 217-5305 *T. rubrum*
HS 214-8900 *T. rubrum*
HS 217-70 *T. rubrum*
HS 214-5152 *T. rubrum*
HS 217-546 *T. rubrum*
CBS 115317 *T. rubrum*
HS 215-28582 *T. rubrum*
CBS 289.86T *T. kanei* (IHEM 26530)
CBS 102856 *T. rubrum*
CBS 287.86 *T. rubrum*
CBS 592.68T *T. fluviomuniense*
Group 3A
HS 217-5111 *T. rubrum*
CBS 191.69 *T. rubrum*
CBS 304.60 *T. rubrum*
CBS 375.92 *T. violaceum*
CBS 319.31 *T. violaceum*
IHEM 10480 *T. violaceum*
Group 1
IHEM 10481 *T. violaceum*
IHEM 13461 *T. violaceum*
IHEM 13462 *T. violaceum*

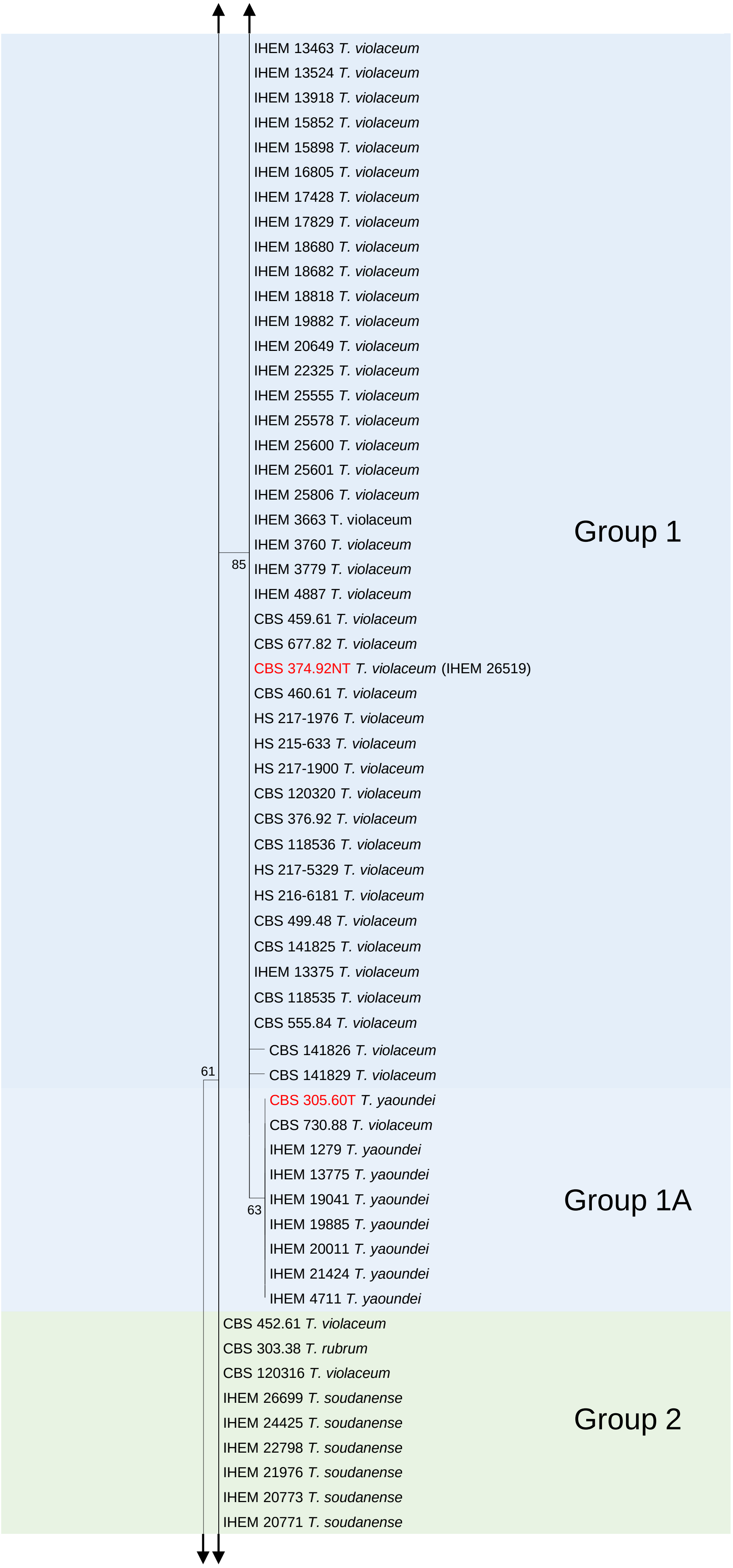
IHEM 13463 T. violaceum
IHEM 13524 T. violaceum
IHEM 13918 T. violaceum
IHEM 15852 T. violaceum
IHEM 15898 T. violaceum
IHEM 16805 T. violaceum
IHEM 17428 T. violaceum
IHEM 17829 T. violaceum
IHEM 18680 T. violaceum
IHEM 18682 T. violaceum
IHEM 18818 T. violaceum
IHEM 19882 T. violaceum
IHEM 20649 T. violaceum
IHEM 22325 T. violaceum
IHEM 25555 T. violaceum
IHEM 25578 T. violaceum
IHEM 25600 T. violaceum
IHEM 25601 T. violaceum
IHEM 25806 T. violaceum
IHEM 3663 T. violaceum
IHEM 3760 T. violaceum
85
IHEM 3779 T. violaceum
IHEM 4887 T. violaceum
CBS 459.61 T. violaceum
CBS 677.82 T. violaceum
CBS 374.92NT T. violaceum (IHEM 26519)
CBS 460.61 T. violaceum
HS 217-1976 T. violaceum
HS 215-633 T. violaceum
HS 217-1900 T. violaceum
CBS 120320 T. violaceum
CBS 376.92 T. violaceum
CBS 118536 T. violaceum
HS 217-5329 T. violaceum
HS 216-6181 T. violaceum
CBS 499.48 T. violaceum
CBS 141825 T. violaceum
IHEM 13375 T. violaceum
CBS 118535 T. violaceum
CBS 555.84 T. violaceum
CBS 141826 T. violaceum
61
CBS 141829 T. violaceum
CBS 305.60T T. yaoundei
CBS 730.88 T. violaceum
IHEM 1279 T. yaoundei
IHEM 13775 T. yaoundei
IHEM 19041 T. yaoundei
63
IHEM 19885 T. yaoundei
IHEM 20011 T. yaoundei
IHEM 21424 T. yaoundei
IHEM 4711 T. yaoundei
CBS 452.61 T. violaceum
CBS 303.38 T. rubrum
CBS 120316 T. violaceum
IHEM 26699 T. soudanense
IHEM 24425 T. soudanense
IHEM 22798 T. soudanense
IHEM 21976 T. soudanense
IHEM 20773 T. soudanense
IHEM 20771 T. soudanense
Group 1
Group 1A
Group 2

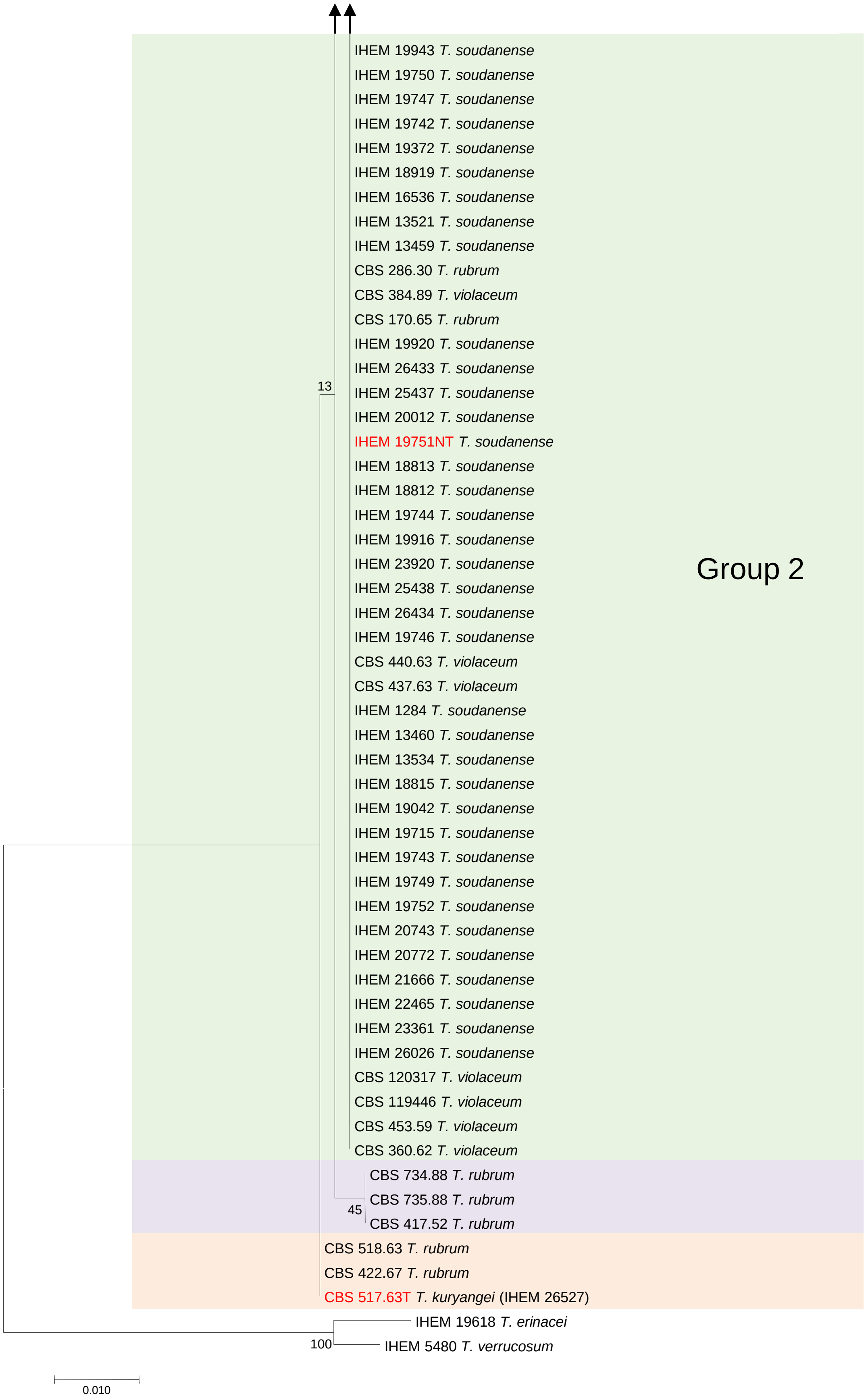
IHEM 19943 T. soudanense
IHEM 19750 T. soudanense
IHEM 19747 T. soudanense
IHEM 19742 T. soudanense
IHEM 19372 T. soudanense
IHEM 18919 T. soudanense
IHEM 16536 T. soudanense
IHEM 13521 T. soudanense
IHEM 13459 T. soudanense
CBS 286.30 T. rubrum
CBS 384.89 T. violaceum
CBS 170.65 T. rubrum
IHEM 19920 T. soudanense
IHEM 26433 T. soudanense
13
IHEM 25437 T. soudanense
IHEM 20012 T. soudanense
IHEM 19751NT T. soudanense
IHEM 18813 T. soudanense
IHEM 18812 T. soudanense
IHEM 19744 T. soudanense
IHEM 19916 T. soudanense
IHEM 23920 T. soudanense
Group 2
IHEM 25438 T. soudanense
IHEM 26434 T. soudanense
IHEM 19746 T. soudanense
CBS 440.63 T. violaceum
CBS 437.63 T. violaceum
IHEM 1284 T. soudanense
IHEM 13460 T. soudanense
IHEM 13534 T. soudanense
IHEM 18815 T. soudanense
IHEM 19042 T. soudanense
IHEM 19715 T. soudanense
IHEM 19743 T. soudanense
IHEM 19749 T. soudanense
IHEM 19752 T. soudanense
IHEM 20743 T. soudanense
IHEM 20772 T. soudanense
IHEM 21666 T. soudanense
IHEM 22465 T. soudanense
IHEM 23361 T. soudanense
IHEM 26026 T. soudanense
CBS 120317 T. violaceum
CBS 119446 T. violaceum
CBS 453.59 T. violaceum
CBS 360.62 T. violaceum
CBS 734.88 T. rubrum
CBS 735.88 T. rubrum
45
CBS 417.52 T. rubrum
CBS 518.63 T. rubrum
CBS 422.67 T. rubrum
CBS 517.63T T. kuryangei (IHEM 26527)
IHEM 19618 T. erinacei
100
IHEM 5480 T. verrucosum
0.010

Figure. S1. Maximum Likelihood tree based on General Time Reversible model. The tree with the highest log likelihood (–1089.87) is shown. The percentage of trees in which the associated taxa clustered together is shown next to the branches. Initial tree(s) for the heuristic search were obtained automatically by applying Neighbor-joining and BioNJ algorithms to a matrix of pairwise distances estimated using the Maximum Composite Likelihood (MCL) approach, and then selecting the topology with superior log likelihood value. The tree is drawn to scale, with branch lengths measured in the number of substitutions per site. The analysis involved 175 nucleotide sequences with 565 positions in the final dataset. Evolutionary analyses were conducted in MEGA7. Names of the isolates in the tree are the original names in CBS and IHEM database.
